# Eleven-Month-Olds Link Sound Properties With Animal Categories

**DOI:** 10.3389/fpsyg.2020.559390

**Published:** 2020-10-19

**Authors:** Ena Vukatana, Michelle S. Zepeda, Nina Anderson, Suzanne Curtin, Susan A. Graham

**Affiliations:** Department of Psychology, University of Calgary, Calgary, AB, Canada

**Keywords:** categorization, category–property links, inductive reasoning, infancy, generalization

## Abstract

We examined 11-month-olds’ tendency to generalize properties to category members, an ability that may contribute to the inductive reasoning abilities observed in later developmental periods. Across three experiments, we tested 11-month-olds’ (*N* = 113) generalization of properties within the cat and dog categories. In each experiment, infants were familiarized to animal–sound pairings (i.e., dog barking; cat meowing) and tested on this association and the generalization of the sound property to new members of the familiarized categories. After familiarization with a single exemplar, 11-month-olds generalized the sound to new category members that were both highly similar and less similar to the familiarized animal (Experiment 1). When familiarized with mismatched animal–sound pairings (Experiment 2; i.e., dog meowing; cat barking), 11-month-olds did not learn or generalize the sound properties, suggesting that infants have pre-existing expectations about the links between the characteristic sound properties and the animal categories. When familiarized with unfamiliar sound–animal pairings (Experiment 3; i.e., dog-unfamiliar sound), 11-month-olds linked the animals with the novel sounds but did not generalize to new category members. Taken together, these findings highlight the conditions under which young infants generalize properties from one exemplar to other category members.

## Introduction

Category-based induction is a critical aspect of human reasoning, allowing individuals to generalize beyond what is known to new instances and situations. Although much is known about inductive reasoning during the late infancy and preschool years (see Gelman, 2003; Hayes et al., 2010; Hayes and Heit, 2013 for reviews), comparatively little research has focused on the emergence of this ability during the first year of life. In these experiments, we examined a fundamental step in category-based inductive reasoning—the ability to establish category–property links and to generalize properties to new category members. More specifically, we investigated 11-month-olds’ tendency to extend a characteristic sound property from one category member to another in the context of the naturally occurring categories of cats and dogs. A large body of research has demonstrated that infants and preschoolers draw upon a variety of cues when making inductive inferences. For example, in the absence of other cues, infants and preschoolers expect perceptually similar objects, particularly those similar in shape, to share common properties (e.g., Gelman and Coley, 1990; Welder and Graham, 2001; Graham et al., 2004; Sloutsky et al., 2007; Graham and Diesendruck, 2010; Graham et al., 2012; Noles and Gelman, 2012; Keates et al., 2014; Switzer and Graham, 2017). Young children will also use information about shared category membership as the basis for their inductive inferences (e.g., Mandler and McDonough, 1996; Poulin-Dubois et al., 2006; Graham et al., 2012; Sweller and Hayes, 2014; Switzer and Graham, 2017). For example, when two objects are labeled with the same count noun, a conventional marker of category membership, 13- to 22-month-olds generalize properties from one category member to another, even when these objects are perceptually dissimilar (Graham and Kilbreath, 2007; Keates and Graham, 2008). Other work has established that infants also draw upon functional object parts when making property inferences [e.g., parts that can help to differentiate between animate and inanimate objects, such as wheels (Rakison, 2005)]. There has been significant debate in the literature around the mechanism that underlies young children’s inductive inferences (e.g., Gelman, 2003; Smith et al., 2003; Smith and Samuelson, 2006; Robinson and Sloutsky, 2007a,b; Sloutsky and Robinson, 2008; Waxman and Gelman, 2009; Deng and Sloutsky, 2012; Graham et al., 2012; Noles and Gelman, 2012; Sloutsky and Fisher, 2012; Sutherland and Cimpian, 2015). Despite this debate, it is clear from the existing literature that young children can flexibly adjust their property extensions depending on the type of cues that are available at the time of learning.

To date, comparatively little is known about the emergence of a key component of category-based inductive reasoning, namely, the ability to establish category–property links and to generalize properties to new category members in infants younger than 12 months of age (although we note that other research has examined general inductive reasoning in young infants, see Dewar and Xu, 2010; Denison et al., 2013). Two early studies have provided some insights into young infants’ ability to make category–property inferences (Baldwin et al., 1993; McDonough and Mandler, 1998). In the first study to address this question, Baldwin et al. (1993) examined 9- to 16-month-olds’ reasoning about the non-obvious properties of artifacts using a generalized imitation paradigm. Specifically, after being presented with a target object that could be acted on to produce an outcome (e.g., a horn that honked when squeezed), an experimenter observed whether infants would attempt to elicit the desired outcome on varying test objects. Results indicated that infants made property generalizations on the basis of perceptual features, expecting only perceptually similar objects to share common properties.

In another seminal study, McDonough and Mandler (1998) used a generalized imitation paradigm to investigate 9- and 11-month-olds’ property extensions about the broad categories of animals and vehicles. Here, infants broadly generalized properties within each domain but restricted their generalizations to within-category members (e.g., infants expected diverse members of the animal category, such as a cat and a bird, but not vehicles, to share the property of *sleeping*). McDonough and Mandler argued that infants’ generalizations are guided by the broad nature of early categories, noting that global-level categories emerge earlier in development than basic-level categories [and thus generalizations are not restricted to basic-level classes at this developmental stage; see Murphy (2016) for a discussion of the validity of this distinction]. Others, however, have demonstrated that infants respond to basic-level distinctions (e.g., Quinn et al., 1993; Behl-Chadha, 1996; Graham et al., 1998), raising the question of whether infants can appropriately restrict their property generalizations within basic-level classes at earlier developmental stages.

The two studies described above suggest that, during the first year of life, infants begin to infer that category members share properties. Yet, as noted, they also leave open several questions regarding the emergence of inductive reasoning. In these studies, we examined a critical precursor of inductive reasoning—*the ability to form associations between categories and their respective properties*. That is, our goal was to examine infants’ tendency to make category–property links, an ability that may be related to the inductive reasoning abilities observed in later developmental periods. In doing so, we aimed to provide new insights into how young infants begin to organize the vast amount of new information they encounter during the first year of life and to examine how drawing upon naturally occurring animal categories may help infants make predictions about the shared properties of category members.

When designing our studies, we took as a starting point the results of research demonstrating that, in the context of *novel*, basic-level animal categories, the ability to establish category–property links may not emerge until 11 months of age (Vukatana et al., 2015; Zepeda and Graham, 2019). Specifically, studies have demonstrated that 11-month-olds, but not 9-month-olds, will generalize a newly learned sound property to new members of a novel animal category. Critically, however, 11-month-olds required the presentation of multiple category exemplars to extend properties to new category members. That is, 11-month-olds do not appear to form associations between *novel* animals and *novel* animal sounds when familiarized with a single exemplar. When considered in the context of McDonough and Mandler’s (1998)’s findings, these results raise an interesting puzzle regarding potential differences in the tendency to establish category–property links during the first year of life. In McDonough and Mandler’s study, infants not only made property extensions by 9 months of age but also did so when presented with a single category exemplar, highlighting that infants may draw upon different cues when making property extensions under those learning conditions.

In the following experiments, we examined the question of whether infants can establish category–property links in the context of naturally occurring animal kinds (i.e., cats and dogs). We focused on the specific categories of cats and dogs for the following reasons. First, infants readily categorize cats and dogs in experimental tasks. That is, several studies to date have demonstrated that infants as young as 3–4 months of age respond to the categorical distinction between cats and dogs (e.g., Quinn et al., 1993; Eimas and Quinn, 1994; Behl-Chadha, 1996; Oakes and Ribar, 2005), even when presented only with silhouettes of these animals (Quinn and Eimas, 1996). Remarkably, infants also recognize the features that can be most informative in distinguishing between cats and dogs (i.e., the head region) and use that information to guide their categorical decisions (Quinn and Eimas, 1996; Quinn et al., 2009). Second, infants are likely to have encountered these naturally occurring categories in their daily life. For many infants, those encounters occur through direct exposure to pets—in fact, a recent survey found that among the 65% of families that own pets in the United States, 53% of them owned cats and 68% owned dogs (American Pet Products Association,, 2016). For other infants, exposure can occur through a variety of means, including toys, books, or television.

When designing our studies, we also drew upon research examining infants’ intermodal matching that has suggested that infants may have some pre-existing expectations about category–property links in the context of highly salient stimuli (e.g., faces, emotional stimuli). For example, studies have shown 5- to 7-month-olds can match voices to faces on the basis of gender (Walker-Andrews et al., 1991; Bahrick et al., 2005), age of speaker (Bahrick et al., 1998), and affect (Walker, 1982; Soken and Pick, 1992; Walker-Andrews, 1997; Vaillant-Molina et al., 2013). More recently, other work has demonstrated that infants will also match affective vocalizations to appropriate body movements (Zieber et al., 2014). The ability to match on the basis of affect also extends to other species. For instance, Flom et al. (2009) demonstrated that 6-month-old infants match aggressive and non-aggressive dog vocalizations to pictures of dogs depicting the respective facial expressions. Taken together, these studies suggest that infants may have some pre-existing expectations about category–property links in the context of highly salient stimuli (e.g., faces, emotional stimuli).

Our studies approached the question of whether infants can establish category–property links from a different direction. In Experiments 1–3, we examined infants’ intermodal associations in the experimental context and, more importantly, the *generalization* of this information. To achieve this goal, we assessed 11-month-olds’ extension of the sound properties associated with the categories of cats and dogs (Experiments 1–3). In these experiments, infants were familiarized with a single exemplar of animal–sound pairings (i.e., a cat and a dog, paired with their characteristic sounds) over a series of trials. Following familiarization, infants’ acquisition of the animal–sound pairing and their ability to generalize the sound property to a new category member were tested. Experiment 1 examined whether infants generalized an animal–sound association both within exemplars representing the same animal breed (e.g., a Labrador retriever to another Labrador retriever) and to another breed (e.g., a Labrador retriever to a Terrier). Experiment 2 focuses on 11-month-olds’ generalization of sound properties when the category-sound pairings were mismatched. That is, infants were familiarized with the opposite animal–sound pairings used in the previous experiment (i.e., cats barking and dogs meowing) to explore whether there were any constraints on the sounds that infants were willing to associate with these animal categories. Finally, in Experiment 3, 11-month-olds were familiarized with cats and dogs paired with novel sounds to further examine whether infants had any prior expectations about the sounds emitted by members of naturally occurring categories.

## Preliminary Experiment

To lay the foundation for our subsequent studies, we first examined whether 11-month-olds would correctly match a characteristic sound with the appropriate animal in the absence of exposure to the animal–sound pairing in the experimental context. Full details on participants, methods, and results can be found in Appendix A. If infants spontaneously link the characteristic sounds that cats and dogs make to their depicted referents, this would suggest that they have already formed a robust category-sound link at this age.

Eleven-month-olds (*n* = 32) were exposed to static pictures of a cat and a dog with no accompanying sound. We then tested infants in a preferential looking paradigm. Videos of a cat and a dog were presented side-by-side while one sound played (either meowing or barking). If infants brought previous knowledge about the sounds emitted by cats and dogs to the experiment, we expected that they would look toward the target animal (i.e., the animal that matched the sound) at rates significantly greater than chance. In other words, consistent with previous work, we expected infants to spend a greater proportion of time looking to the congruent match (e.g., Bahrick et al., 1998; Oakes and Madole, 2000; Flom et al., 2009).

Results indicated that 11-month-olds did not show evidence of spontaneously matching the respective sounds to cats and dogs under the specific conditions of our experimental task, suggesting that some familiarization with the animal–sound pairing may be required to activate infants’ representations of these naturally occurring animals. This outcome provides the foundation for the following experiments in which we consider infants’ generalization of animal–sound mappings, following familiarization with the animal–sound mapping in the experimental context.

## Experiment 1

The goal of Experiment 1 was to examine 11-month-olds’ ability to generalize the sound properties of cats and dogs to new category members, when familiarized with a single exemplar. We familiarized infants with two animal–sound pairings (one cat and one dog making their respective characteristic sounds) and then tested them in one of two conditions—the *same breed* condition and the *different breed* condition. Within each condition, infants were presented with two types of test trials. To evaluate the acquisition of the animal–sound mapping, *same* trials entailed the side-by-side presentation of the same two animals observed during familiarization, accompanied by one of the characteristic sounds (i.e., meowing or barking). *Extension* trials assessed infants’ ability to extend the sound property to new category members and differed across conditions. In the *same breed* condition, infants saw new cat and dog exemplars that differed only in color from their counterparts during familiarization while one sound played. In this condition, we aimed to determine whether infants could make property generalizations to highly similar category members. In the *different breed* condition, infants were presented with new, less perceptually similar category members, reflecting a change in breed. By examining infants’ generalization to less perceptually similar category members, we sought a more robust examination of infants’ ability to establish category-property links, as opposed to solely shaped-based associations. This question is critical, given that despite sharing a number of key attributes [e.g., parts, shape, and texture; as identified by Rosch et al. (1976)], category members do not typically differ from one another solely in terms of color. We expected that, if infants formed an animal–sound mapping and generalized the sound property to new category members, their proportion of looking to the target animal would be significantly greater than chance for both *same* and *extension* trials.

### Method

#### Participants

The final sample consisted of 53 eleven-month-olds, randomly assigned to either the *same breed condition* (*N* = 26) or the *different breed condition* (*N* = 27). Sixteen additional infants were tested but excluded for the following reasons: did not complete the experiment (*n* = 5); experimenter error (*n* = 5); parental interference (*n* = 1); excessive fussiness (video could not be coded due to infant’s behavior; *n* = 1); failure to look at both animals for more than 1 s during test trials (*n* = 3); and preference for one animal (defined as looking more than 70% to the same animal across test trials; *n* = 1). Assuming β of 0.80 and a two-tailed p value of 0.05, a power analysis indicated that a sample size of 26 has sufficient power to detect a Cohen-defined medium effect size of *d* = 0.57 (an effect size in keeping with previous research using a similar paradigm). Infants came from homes in which English was the predominant language spoken, and although not formally assessed, infants were primarily of European descent. The majority of parents (84%) had achieved some level of postsecondary education. Information on mean age, gender, and whether infants had a pet at home is included in Table 1. This experiment was approved by the Conjoint Faculties Research Board at the University of Calgary (Project title: Reasoning about object categories and properties during early childhood REB16-0423).

**TABLE 1:** Demographic information as a function of experiment.

	Expt. 1		Expt. 2	Expt. 3
			
	Same breed (*n* = 26)	Different breed (*n* = 27)	Incongruent sound (*n* = 30)	Unfamiliar sound (*n* = 30)
Age*				
Mean (*SD*) Range	11.37 (0.27) 11.05–11.93	11.43 (0.24) 11.02–11.93	11.49 (0.28) 11.08–11.93	11.46 (0.27) 11.02–11.97
Gender	11 girls 15 boys	14 girls 13 boys	12 girls 18 boys	13 girls 17 boys
Have pets at home	3—dog 9—both 14—no pets	5—dog 2—cat 6—both 14—no pets	3—dog 7—cat 9—both 11—no pets	5—dog 4—cat 7—both 14—no pets

*Age in months.

#### Stimuli

**FIGURE 1:**
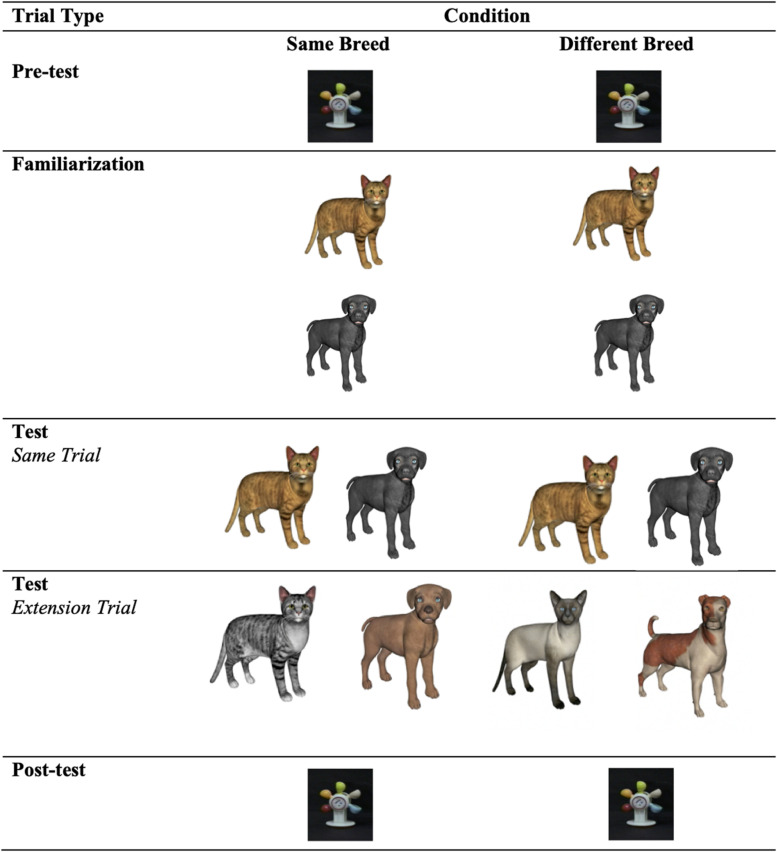
The sounds paired with the dogs and cats during familiarization varied as a function of Experiment. In Expt. 1, cats were paired with their characteristic “meow” and dogs were paired with their characteristic “bark.” In Expt. 2, animals were paired with incongruent sounds (i.e., cats were paired with barking sounds and the dogs were paired with meowing sounds). In Expt. 3, each animal was paired with a distinctive novel animal sound.

During the pre- and posttest trials, infants were presented with a waterwheel accompanied by music. The visual stimuli presented during familiarization and at test consisted of animations of naturally occuring animals (i.e., cats and dogs), accompanied by their respective category sounds (i.e., *meowing* and *barking*). See Figure 1. Two exemplars from each animal category that were highly similar, differing only in only color from one another, were used in the *same breed condition*, and two exemplars that were less perceptually similar to one another (i.e., two cats and two dogs of different breeds) were used in the *different breed condition*. The sound stimuli were two real animal sounds: meowing (76.24 dB and 576.46 Hz) and barking (75.96 dB and 600.02 Hz). The mouth movements of the animals and the sound were synchronous, in line with research suggesting that infants learn arbitrary relations between visual and auditory stimuli when they are presented in synchrony (e.g., Bahrick et al., 2005).

#### Apparatus

Infants were tested in a soundproof, dimly lit room and sat on their parent’s lap or on a highchair. The visual stimuli were presented on a 122 cm × 91.5 cm monitor, and the auditory stimuli were played from a speaker placed directly above the monitor. Parents listened to music through headphones and were asked to have minimal interaction with their infants. The Habit X 1.0 program was used to run the experiment (Cohen et al., 2004). All testing sessions were recorded for later coding of infants’ looking time on a frame-by-frame basis.

#### Design and Procedure

Prior to the experimental task, parents were asked about infants’ exposure to cats and dogs, as research has suggested that infants’ prior experience may impact their performance on laboratory tasks (Kovack-Lesh et al., 2008, 2014; Träuble et al., 2009). Although the majority of infants in our studies had exposure to cats and dogs (through direct contact and/or through books, TV, or toys), we choose to more precisely characterize infants’ experience. Specifically, we asked whether infants had a pet at home, whether family friends or members had pets that the infant had regular (i.e., weekly) contact with, and the amount of weekly exposure that infants had to cats and/or dogs overall (in hours). Due to the small sample sizes and exploratory nature of analyses linking pet experience with performance on our experimental task, we only report the results of analyses of these measures in S2 Appendix B.

Infants were tested in either the *same breed* condition or the *different breed* condition. See Figure 1 for an overview of the design. The experimental task began with a pretest trial and ended with a posttest trial, both of which involved the presentation of a waterwheel accompanied by music. The pre- and posttest trials lasted 20 s each. The pretest was followed by the familiarization phase and then the test phase.

The familiarization phase was identical across the two conditions. Infants were familiarized to two animal–sound pairings presented sequentially (e.g., Orange Cat–*Meow* and Black Dog–*Bark*) for a total of 24 trials. The familiarization phase consisted of six blocks of four trials each; each animal was presented twice within a given block. We counterbalanced the order in which the animals appeared within and across blocks as well as the exemplars used for familiarization. Each familiarization trial lasted 10 s. During each trial, infants observed the animal standing in profile (2 s) and turning its head to face the front (2 s). Once the animal was facing the infants, it opened its mouth to produce a sound, alternating between sound (0.5 s) and silence (1 s) for the remaining 6 s of the trial.

For the test phase, infants were tested using a preferential looking paradigm. We opted to use a preferential looking paradigm at test as opposed to a switch paradigm, given that this procedure may place fewer cognitive demands on infants (e.g., Yoshida et al., 2009). For example, there is evidence demonstrating that, when asked to learn an association between an auditory and visual stimulus, infants show evidence of learning when tested in a preferential looking paradigm (e.g., Yoshida et al., 2009) but not when tested in a switch task (e.g., Werker et al., 2002; Vukatana et al., 2015). These findings have been interpreted to suggest that determining the “best fit” between the stimuli is easier than determining a mismatch (e.g., Yoshida et al., 2009). Given the complexity of tracking two separate animals and two separate sounds in our experiments, we used a preferential looking paradigm in an attempt to facilitate infants’ ability to demonstrate their learning.

Infants were tested with two types of test trials, during which videos of a cat and a dog were presented side by side (53 cm apart) while infants heard one sound (barking or meowing). On these trials, the animals faced the infants and opened and closed their mouths in synchrony for a total of 20 s, alternating between periods of sound (0.5 s) and silence (1 s). *Same* trials involved the presentation of the two familiarized animals while one sound played (e.g., Orange Cat–Black Dog–Bark). *Extension* trials differed depending on the condition. In the *same breed* condition, infants were presented with a new exemplar in a new color from each respective category. In the *different breed* condition, infants were tested with a new, less perceptually similar category member, reflecting a change in breed. The test trials were always presented in the following order: same, extension, same, extension. We aimed to ensure that infants had formed an animal–sound mapping during familiarization, prior to asking them to generalize the sound property to a new category member. This is in line with previous research, suggesting that infants benefit from exposure to a familiar event prior to being asked to generalize information to new exemplars (e.g., McDonough and Mandler, 1998; Haryu et al., 2011).

Looking times for all trials were coded on a frame-by-frame basis from the video to obtain a more accurate measure of infants’ looking times as compared to online coding. Center fixations were coded for the familiarization trials, and left and right looks were coded for the test trials. Coders were unaware of the study purpose and hypotheses and were unable to identify the target animal during test trials (as coding was conducted with the sound turned off). Inter-rater reliability for 20% of the data (*n* = 11) was high (ICC = 0.99, *p* < 0.001).

### Results and Discussion

To rule out any potential role of fatigue, our first set of analyses focused on infants’ looking time to the pretest, first and last block of familiarization, and posttest trials. Because the pre- and posttest trials (20 s) differed in length from the familiarization trials (10 s), we first calculated a proportion of looking score for each type of trial (i.e., we divided infants’ looking time during each trial by the total trial length; see Table 2 for non-proportioned looking times). A 2 (condition—*same breed* vs. *different breed*) × 4 (trial type—*pretest*, *first block of familiarization*, *last block of familiarization*, and *posttest*) mixed-model ANOVA revealed a significant main effect of trial type, *F*(3,153) = 33.37, *p* < 0.001, $\eta_{\text{p}}^{2}$ = 0.40. Planned pairwise comparisons indicated that infants’ proportion of looking time to the pretest (*M* = 0.90, *SD* = 0.11) did not differ from their proportion of looking time to the posttest (*M* = 0.90, *SD* = 0.13; *p* = 0.810). As expected, infants’ looking time decreased over the course of familiarization, as infants spent a greater proportion of time looking during the first block of familiarization (*M* = 0.96, *SD* = 0.07) compared to the last block of familiarization (*M* = 0.75, *SD* = 0.17; *p* < 0.001). Finally, infants recovered their looking following familiarization, as their proportion of looking to the posttest was significantly higher than their proportion of looking to the last block of familiarization (*p* < 0.001). All other effects were non-significant (*p*s > 0.220).

**TABLE 2:** Mean looking time for pre-test, post-test, and first and last familiarization block as a function of experiment.

	Pre-test [mean (*SD*)]	First block of familiarization	Last block of familiarization	Post-test
Experiment 1				
Same breed	18.54 (1.37)	9.78 (0.38)	7.50 (1.50)	17.91 (2.40)
Different breed	17.35 (2.66)	9.34 (0.93)	7.59 (1.82)	18.15 (2.66)
Experiment 2	17.73 (2.31)	9.78 (0.50)	7.51 (1.49)	18.27 (2.76)
Experiment 3	18.24 (2.26)	9.77 (0.52)	8.08 (1.61)	18.02 (2.50)

Our primary analyses of interest focused on 11-month-olds’ looking time to the target animal during test trials. Recall that if infants matched each sound to its respective animal, we expected their proportion of looking to the target animal to be significantly greater than chance. As a first step, we calculated a proportion of looking time for *same* and *extension* trials by dividing infants’ looking time to the target animal by their total looking time during a given trial. A 2 (condition—*same breed* vs. *different breed*) × 2 (trial type—*same* and *extension*) × 2 (order—*first* vs. *second block of trials*) mixed-model ANOVA yielded no significant main effects or interactions (*p*s > 0.073).

**FIGURE 2:**
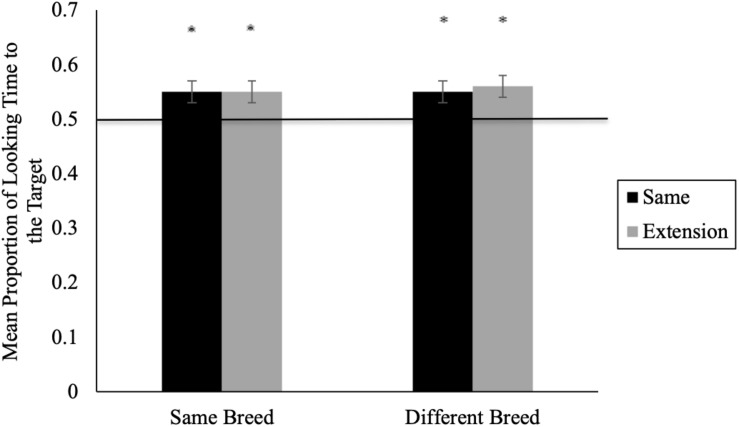
11-month-olds’ mean proportion looking time during same and extension trials for Experiment 1 (*^∗^p* < 0.05). Error bars represent standard error.

Our key analyses examined whether infants’ looking toward the target was significantly different from chance (i.e., whether infants matched the sound to its respective animal). Here, we averaged across trial blocks to obtain a mean proportion of looking for *same* and *extension* trials (given the non-significant order effect) and compared infants’ performance within each condition to chance levels (i.e., 0.50) using two-tailed *t*-tests. See Figure 2 for mean proportion looking time during same and extension trials. In the *same breed* condition, 11-month-olds’ proportion of looking to the target animal was significantly greater than chance for *same* (*M* = 0.55, *SD* = 0.10) and *extension* (*M* = 0.55, *SD* = 0.11) trials, *t*(25) = 2.35, *p* = 0.027, *d* = 0.47 and *t*(25) = 2.44, *p* = 0.022, *d* = 0.49, respectively (see Figure 2). Similarly, in the *different breed* condition, 11-month-olds’ proportion of looking to the target animal was significantly greater than chance for both *same* (*M* = 0.55; *SD* = 0.11), *t*(26) = 2.34, *p* = 0.028, *d* = 0.45 and *extension* (*M* = 0.56, *SD* = 0.08), *t*(26) = 3.58, *p* < 0.001, *d* = 0.69 trials.

To complement the above analyses, we used a Bayes Factor analysis to assess the relative strength of the evidence for the null hypothesis versus the alternative hypothesis [i.e., looking to the matching animals at above chance levels (Dienes, 2014)]. To compute our Bayes Factors (BF), we used the online Java-based calculator developed by Anupam Singh^1^ and modeled the alternative hypothesis (H1) by using the relevant mean difference from Vukatana et al. (2015) (*M* = 0.09; Experiment 2). In that experiment, this mean difference signaled above chance matching of the target sound to the target animal. We used a half-normal or one-tailed parameter as our prediction was directional; we predicted that infants’ proportion of looking to the target animal would be significantly greater than chance. To interpret our Bayes Factor values, we adhered to the interpretive conventions outlined by Dienes (2014): that is, a BF of 3 or above indicates substantial evidence for the alternative hypothesis over the null hypothesis, while a BF of 1/3 or below is considered to indicate substantial evidence for the null over than alternative hypothesis (2014). Using the calculator, we computed Bayes Factors for *same* and *extension* trials for each condition. In the *same breed* condition, the Bayes Factors provided support for the alternative hypothesis for both *same* and *extension* trials [*B*_*H(*0,0.09)_ = 5.97 and *B*_*H* (0,0.09)_ = 7.78, respectively]. This pattern held for infants’ performance in the *different breed* condition [*B*_*H* (0,0.09)_ = 5.89 for *same* trials and *B*_*H* (0,0.09)_ = 17.7 for *extension* trials], suggesting that infants learned and generalized the sound property.

The results of Experiment 1 indicated that, across conditions, infants learned the animal–sound mapping and generalized the sound property to new category members, indicating that 11-month-olds incorporated objects properties into their categories of cats and dogs when familiarized with the animal–sound pairing in the experimental context.

## Experiment 2

Our findings in Experiment 1 indicated that 11-month-olds readily generalize characteristic sound properties of dogs and cats to new category exemplars after presentation with a single category member. These findings contrast those of research examining infants’ property extensions in the context of unfamiliar animate kinds (Vukatana et al., 2015; Zepeda and Graham, 2019). Across those studies, 11-month-olds successfully generalized a sound property to highly similar category members (i.e., new exemplars in a new color) when presented with multiple exemplars of a category but not when presented with a single category exemplar. Our findings raise the possibility that infants may have been drawing upon their *pre-existing representations* for dogs and cats in the current studies, which were activated by familiarization with the sound–animal pairings during the familiarization phase, given that cats and dogs represent naturally occurring categories. Note that it is possible that infants have pre-existing visual representations of the animals but learned the sound–animal mapping during the familiarization period or that infants have the sound–animal mapping but required some familiarization in the experimental context to activate that representation.

In Experiment 2, we examined the extent to which infants’ pre-existing representations may facilitate or restrict learning by exploring whether 11-month-olds would associate incongruent sound properties with cats and dogs. Specifically, we asked whether familiarization with a single exemplar would lead 11-month-olds to associate dogs with meowing and cats with barking. In testing infants’ learning of incongruent sounds, we sought to determine whether infants’ willingness to learn and generalize sound properties is constrained to naturally occurring pairings (i.e., cats meowing and dogs barking) or whether infants can flexibly incorporate any sound property into their representations.

In this experiment, we tested 11-month-olds in similar conditions to the same breed condition in Experiment 1. Instead of presenting the animals paired with their typical category sounds, children were familiarized with mismatched animal–sound pairings (i.e., a single exemplar of a cat barking and a dog meowing). Here, we posited that, if infants were to learn and generalize the incongruent sounds, the findings would provide evidence for infants’ flexible animal–sound representations (i.e., would suggest that those pre-existing representations are not necessarily constrained to naturally occurring animal–sound pairs). On the contrary, if infants fail to learn and generalize the incongruent sounds, the results would provide support for the view that infants may draw upon pre-existing representations and thus restrict their mappings and generalizations to naturally occurring pairings.

### Method

#### Participants

Thirty 11-month-olds were included in the final sample and recruited from the same population as the previous experiment. An additional 15 infants were tested but excluded from analysis for the following reasons: did not complete experiment (*n* = 4); experimenter error (*n* = 2); parent interference (*n* = 4); failure to look at both animals for more than 1 s during test trials (*n* = 5). Assuming β of 0.80 and a two-tailed *p*-value of 0.05, a power analysis indicated that a sample size of 26 has sufficient power to detect a Cohen-defined medium effect size of *d* = 0.52. Demographic information is included in Table 1.

#### Stimuli and Apparatus

As in previous experiments, infants were presented with a waterwheel accompanied by music during the pre- and posttest trials. The visual stimuli presented during familiarization were identical to those used for the same breed conditions in Experiment 1 and consisted of animated cats and dogs. The sound stimuli were identical to those used in the previous experiments; however, they were paired with the opposite animals (i.e., a cat was paired with a barking sound and a dog was paired with a meowing sound). During *same* trials, infants were presented with two videos of the animals that they had seen during familiarization presented in synchrony with one of the animal sounds (i.e., barking or meowing). In *extension* trials, infants were presented with an exemplar from each animal category that differed in color from those they had seen during familiarization and one of the animal sounds.

#### Procedure

An overview of the experimental design can be seen in Figure 1. The procedure followed the same format as the same breed condition in Experiment 1. Center fixations were coded for pretest, posttest, and first and last block of familiarization, while left and right looks were coded for test trials. Inter-rater reliability for 20% of the data (*n* = 6) was high (ICC = 0.98, *p* < 0.001).

### Results and Discussion

See Table 2 for non-proportioned looking times for pretest, posttest, and familiarization trials. A within-subjects ANOVA revealed a main effect of trial type, *F*(3,87) = 27.10, *p* < 0.001, $\eta_{\text{p}}^{2}$ = 0.48. As in previous experiments, infants’ proportion of looking to the pretest (*M* = 0.89, *SD* = 0.12) did not differ from their proportion of looking to the posttest (*M* = 0.91; *SD* = 0.14; *p* = 0.32). Infants spent a significantly greater proportion of time looking to the posttest trials than to the last block of familiarization (*M* = 0.75, *SD* = 0.15), indicating that they recovered their looking following familiarization (*p* < 0.001). Finally, infants decreased their looking from the first block (*M* = 0.97; *SD* = 0.05) to the last block of familiarization (*p* < 0.001).

A 2 (trial type—*same* and *extension*) × 2 (order—*first block* vs. *second block* of trials) repeated measures ANOVA yielded only a significant main effect of trial order, *F*(1,29) = 10.64, *p* = 0.003, $\eta_{\text{p}}^{2}$ = 0.27. This main effect indicated that infants looked longer at the target during the first block of trials (*M* = 0.52, *SD* = 0.06), collapsed across trial type, than during the second block of trials (*M* = 0.45, *SD* = 0.09). There were no other significant effects or interactions (*p*s > 0.81). The order effect suggests that, during the second block of trials, infants looked longer to the non-target animal (i.e., matched meowing with the cat rather than matching based on the incongruent pairing presented during familiarization).

**FIGURE 3:**
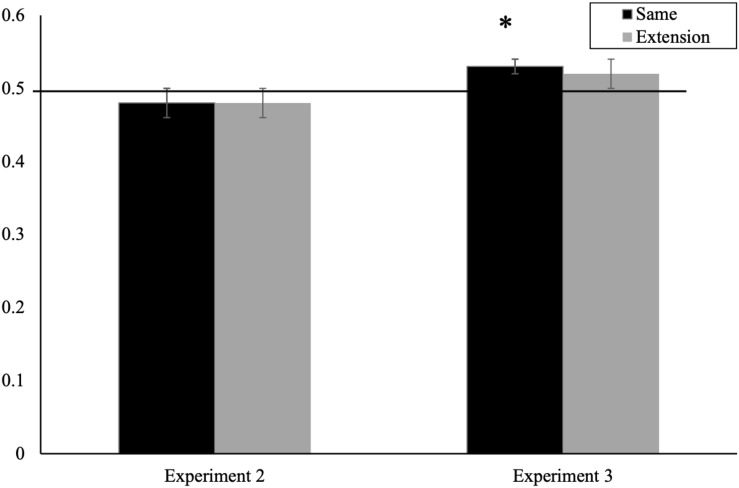
Mean proportion looking time during same and extension trials for Experiment 2 and Experiment 3 (^∗^significantly greater than chance, *p* < 0.05). Error bars represent standard error.

As in the previous experiment, our key analyses focus on whether infants’ looking to the target differed from chance. See Figure 3 for mean proportion of looking as a function of trial. Eleven-month-olds’ proportion of looking to the target animal did not differ from chance for the *same* trials (*M* = 0.48, *SD* = 0.09), *t*(29) = 1.29, *p* = 0.205, *d* = 0.23, or for *extension* trials (*M* = 0.48, *SD* = 0.08), *t*(29) = 1.06, *p* = 0.297, *d* = 0.20. Because of the order effect in the ANOVA, we also did chance-level comparisons for the same and extension trials for each trial block separately. Consistent with the averaged analyses, these values did not differ from chance (*p*s > 0.053). Bayes Factor analyses, using the same parameters and model specified in Experiment 1, supported this conclusion, providing substantial evidence for the null hypothesis for both *same* and *extension* trials [*B*_*H* (0,0.09)_ = 0.08 and *B*_*H* (0,0.09)_ = 0.082, respectively].

Together, these findings indicate that when infants were presented with single exemplars of incongruent naturally occurring animal pairings (i.e., a dog meowing or a cat barking), 11-month-olds did not learn the original animal–sound pairings and did not extend the incongruent sound properties to novel category exemplars. Together, this suggests that infants have pre-existing expectations about the links between the characteristic sound properties and the animal categories, which may restrict their learning of incongruent associations.

## Experiment 3

The results of Experiment 2 suggest that infants did not learn or extend the incongruent sound properties. As such, the pattern of results suggests that infants’ pre-existing knowledge of animal–sound pairings may have guided their performance in the experimental context (in this case, by restricting the animal–sound mappings to those that occur in the real world). In Experiment 3, we aimed to further explore the flexibility of infants’ animal–sound mappings when familiarized with a single exemplar of a category. Specifically, we asked whether infants would learn to associate and generalize novel sounds paired with cats and dogs. In this experiment, we tested 11-month-olds in similar conditions to the same breed condition of Experiment 1 and that of Experiment 2. We familiarized infants with novel sounds paired with the familiar animals (i.e., a single exemplar of a cat paired with an unfamiliar animal sound and a dog paired with a different unfamiliar animal sound). Our predictions stemmed from the results of Experiment 2. That is, if infants restrict their learning and generalization based on naturally occurring animal–sound pairings, then their animal–sound mappings may reflect only those that are represented in the real world. As such, we might expect infants’ looking to not differ from chance for both *same* and *extension* trials.

### Method

#### Participants

Thirty 11-month-olds were included in the final sample and recruited from the same population as the previous experiments. An additional 23 infants were tested but excluded from analysis for the following reasons: did not complete experiment (*n* = 3); experimenter error (*n* = 2); parent interference (*n* = 1); failure to look at both animals for more than 1 s during test trials (*n* = 5); showing a preference for one animal (>70% of the time; *n* = 8); outlier on posttest (*n* = 4). Assuming β of 0.80 and a two-tailed *p*-value of 0.05, a power analysis indicated that a sample size of 26 has sufficient power to detect a Cohen-defined medium effect size of *d* = 0.52. Demographic information is included in Table 1.

#### Stimuli and Apparatus

As in previous experiments, infants were presented with a waterwheel accompanied by music during the pre- and posttest trials. The visual stimuli presented during familiarization were identical to those used for the *same breed* conditions in Experiments 1 and 2 and consisted of animated cats and dogs. The auditory stimuli included a sea lion sound (81.80 dB and 384.45 HZ; obtained from the SeaWorld online sound library) and a rhesus monkey sound (83.17 dB and 428.79 Hz; courtesy of A. Vouloumanos; see Vukatana et al., 2015 for waveforms and spectrograms). During *same* trials, infants were presented with the two animals seen during familiarization, and during *extension* trials, infants were presented with an exemplar from each animal category that differed only in color from those they had seen during familiarization.

#### Procedure

An overview of the experimental design can be seen in Figure 1. The procedure followed the same format as the same breed condition in Experiments 1 and 2, with one exception: novel sounds were paired with the cat and dog stimuli during familiarization. Center fixations were coded for pretest, posttest, and first and last block of familiarization, while left and right looks were coded for test trials. Inter-rater reliability for 20% of the data (*n* = 6) was high (ICC = 0.99, *p* < 0.001).

### Results and Discussion

See Table 2 for non-proportioned looking times for pretest, posttest, and familiarization trials. A within-subjects ANOVA revealed a main effect of trial type, *F*(3,90) = 12.174, *p* < 0.001, $\eta_{\text{p}}^{2}$ = 0.296. As in previous experiments, infants’ proportion of looking to the pretest (*M* = 0.91, *SD* = 0.12) did not differ from their proportion of looking to the posttest (*M* = 0.91; *SD* = 0.12; *p* = 0.951). Infants’ looking to the posttest trials was significantly greater than that to the last block of familiarization (*M* = 0.80, *SD* = 0.17), indicating that they recovered their looking following familiarization (*p* = 0.008). Importantly, infants decreased their looking from the first block (*M* = 0.97; *SD* = 0.05) to last block of familiarization (*p* < 0.001).

As in the previous experiments, our key analyses focus on the test trials and comparisons to chance. A 2 (trial type—*same* and *extension*) × 2 (order—*first block* vs. *second block* of trials) repeated measures ANOVA resulted in no significant main effects or interactions (*p*s > 0.60). As in previous experiments, we then compared whether infants’ looking to the target differed from chance levels. See Figure 3 for mean proportion of looking as a function of trial. Eleven-month-olds’ proportion of looking to the target animal was significantly greater than chance for the *same* trials (*M* = 0.53, *SD* = 0.07), *t*(29) = 2.259, *p* = 0.032, *d* = 0.43 but not on the *extension* trials (*M* = 0.517, *SD* = 0.12), *t*(29) = 0.815, *p* = 0.422, *d* = 0.17. Bayes Factor analyses carried out using the same procedures and parameters used in Experiment 1 generally supported this conclusion, providing substantial evidence for the alternative hypothesis for the *same trial* [*B*_*H* (0,0.09)_ = 3.570] but indeterminate evidence for the extension trial and *B*_*H* (0,0.09)_ = 0.494.

Together, these findings provide insights into the way that infants may use their pre-existing knowledge of animal–sound mappings. Specifically, the results provide tentative support that, when presented with novel animal sounds, infants may associate an atypical sound property with a particular member of a category, suggesting that they may be open to a one-to-one mapping. This may be the case because the presented sound is not associated with the opposing animal category being shown in the experiment nor is it associated with another readily recalled animal. Infants, however, are less likely to generalize that atypical sound to another category member (although we note here that the Bayes analyses do not provide clear support for the null hypothesis on extension trials).

## Cross-Experiment Comparisons

To examine directly whether infants’ tendency to extend sound properties varied across experiments, we carried out two one-way ANOVAs with Experiment as the between-subjects variable: one comparing performance on the Same trials and one comparing performance on the Extension trials. We performed these analyses separately, as the comparisons to chance reported for Experiment 3 suggest differences between the Same and Extension trials. We note that we interpret the results of these comparisons with caution as the experiments were conducted sequentially, and thus, infants were not randomly assigned to experiment.

Comparison of infants’ proportion of looking on the *same* trials across the three experiments indicated a main effect of Experiment, *F*(2,110) = 5.322, *p* = 0.006, $\eta_{\text{p}}^{2}$ = 0.09. *Post hoc* pair-wise comparisons indicated that infants’ proportion of looking to the target animal in Experiment 1 was significantly higher than in Experiment 2 (incongruent sound), *p* = 0.002, but not significantly different from that of Experiment 3 (novel sound), *p* = 0.425. Looking to the target animal on Same trials was significantly higher in Experiment 3 (novel sound) compared to Experiment 2 (incongruent sound), *p* = 0.033.

Comparison of infants’ proportion of looking on the *extension* trials across the three experiments also indicated a main effect of Experiment, *F*(2,110) = 5.125, *p* = 0.007, $\eta_{\text{p}}^{2}$ = 0.09. *Post hoc* pair-wise comparisons indicated that infants’ proportion of looking to the target animal in Experiment 1 was significantly higher than in Experiment 2 (incongruent sound), *p* = 0.002, but not significantly different from that in Experiment 3 (novel sound), *p* = 0.096. Looking to the target animal on *extension* trials was not significantly different in Experiment 3 (novel sound) compared to Experiment 2 (incongruent sound), *p* = 0.197.

Although these results need to be interpreted cautiously for reasons described above, these analyses do suggest that infants performed significantly differently across experiments as a function of the type of association presented.

## General Discussion

Here, we investigated a fundamental step that may be related to later inductive reasoning, namely, infants’ abilities to link properties with categories during the first year of life, within the context of basic-level naturally occurring animals. To begin our investigation, we demonstrated that 11-month-olds generalized properties to new members of the category. Specifically, in Experiment 1, after familiarization with only one member from a naturally occurring animal category, 11-month-olds readily generalized to both highly similar and less similar category members. The results of Experiments 2 and 3 provided insights into how 11-month-olds may use their pre-existing knowledge about the category–property links to guide their learning in the experimental context. Our results demonstrated that, when familiarized with incongruent animal–sound pairings, infants did not learn or generalize the animal–sound mappings. However, when familiarized with novel animal–sound mappings, we found tentative support that infants showed some evidence of learning but not generalizing the sound property (although we note the indeterminate evidence for the null hypothesis on the extension trial in Experiment 3). Taken together, our results provide insights into the developmental origins of the ability to link properties with categories and the information that infants draw upon when extending sound properties.

First, our results add to our understanding of the *conditions* under which infants establish category–property links during the first year of life. That is, our results extend the findings of early research documenting that infants can generalize properties to new category members in the context of artifacts (Baldwin et al., 1993) and global-level categories (McDonough and Mandler, 1998), when presented with only single exemplars of each category. In our studies, we demonstrated that 11-month-olds could also make property extensions in the context of the basic-level, naturally occurring categories of cats and dogs. We note that consideration of the average proportion of looking time to the target in conditions where infants were successful suggests that this ability appears to be emergent during this developmental period. That is, infants’ average proportion of looking time, although above chance and supported by Bayes Factor analyses, landed between 53 and 55%. This may be due to the challenging nature of our task. That is, infants were required to compare the novel exemplar presented at test to the previously encountered familiarization animal, detect similarities between the two, decide that the novel exemplar belongs to the same category, track information about the sound property during familiarization, link the property to a broader category, and decide that the exemplar also shares that property.

Second, our findings that infants generalized to both highly similar and less similar category members, after familiarization with a single exemplar of a category, adds to our understanding of how different types of categories affect infants’ extension of category properties. That is, our findings contrast with those of recent research examining novel animate categories using a very similar paradigm and infants drawn from the same population (Vukatana et al., 2015; Zepeda and Graham, 2019). In those studies, 11-month-olds successfully generalized a sound property to highly similar category members (i.e., new exemplars in a new color) when presented with multiple exemplars of a category but not when presented with a single category exemplar. The differences in infants’ performance across these studies may be attributed to differences in the nature of the stimuli (i.e., novel vs. naturally occurring animals) and infants’ pre-existing knowledge. That is, when faced with *unfamiliar* animate kinds, infants must form their categorical representations and associate the relevant properties with these categories *online*. In order to move beyond a focus on individual exemplars and to establish category–property links, infants appear to require the presentation of multiple exemplars even at 11 months of age (as in Vukatana et al., 2015). In contrast, the current results suggest that the presentation of naturally occurring kinds facilitated infants’ learning and generalization of congruent sounds properties with exposure to only one category member during familiarization.

Why might naturally occurring categories facilitate infants’ category–property generalizations? We note that this discussion is necessarily speculative given that we did not directly contrast naturally occurring versus novel animals in this set of experiments. However, given the similarity in experimental procedures, we posit the following. First, when presented with naturally occurring kinds (i.e., cats and dogs), it may be that infants activated their *pre-existing representations* for these stimuli during the familiarization phase that presented the animal–sound mappings. These representations may have been developed through either direct or indirect exposure to these animals and may reflect perceptual or conceptual learning. That is, we note that our findings are theoretically silent on the specific nature of infants’ pre-existing representations. That is, it is unclear whether infants are activating their conceptual understanding of the categories of cats and dogs or similarity-based representations wherein similar-looking items activate overlapping mental representations (e.g., Mareschal et al., 2000). Whatever the nature of these representations, their activation may have led infants to make property extensions under more difficult testing conditions (i.e., when presented with a single category exemplar and when asked to generalize to less perceptually similar category members of a different breed). Infants did not spontaneously match *meowing* and *barking* to the respective categories of cats and dogs under the learning conditions described in our supplemental experiment (i.e., without familiarization of this animal–sound pairing in the experimental context). Thus, it appears that the familiarization phase served as a reminder of the animal–sound pairing, allowing 11-month-old infants to learn and generalize the sound properties of cats and dogs.

More importantly, the results of Experiments 2 and 3 provide insights into *how* infants pre-existing knowledge of animal–sound pairings may facilitate or restrict their learning in the experimental context. We first demonstrated that, when asked to learn and generalize incongruent sounds, 11-month-olds did not learn or generalize the sound property. That is, familiarization with incongruent animal–sounds pairings inhibited infants’ learning and generalization of the sound property. Interestingly, however, infants demonstrated a different pattern of responding when familiarized with novel sounds paired with cats and dogs. In this case, we found that infants learned the animal–sound pairing presented during familiarization but did not generalize the sound to new category members. As such, our findings suggest that infants may associate the novel sound with a particular member of the category (i.e., formed a one-to-one mapping) when provided with evidence that the specific member produces a sound that may not be typically associated with the broader category (in our case, evidence for this link was provided during the familiarization phase). Importantly, however, infants may inhibit their generalizations of a novel sound to new category members, providing additional evidence for the view that the pre-existing *animal–sound representation* may be guiding infants’ performance in our task. Future research directly contrasting infants’ category–property generalizations with naturally occurring and novel animals within the same set of studies will shed more light on how different types of categories affect infants’ extension of category properties.

Overall, our results suggest that infants may draw upon their general prior exposure to cats and dogs as well as online familiarization to generalize properties to new category members. Our findings, however, also suggest a distinction between the effects of *exposure* to cats and dogs and *specific experience* with these animals. That is, we distinguish between broad exposure to cats and dogs through a variety of means (e.g., direct experience, books, toys, television) and specific experience that focuses solely on direct experience with a pet. Our supplementary analyses indicated that infants’ performance in our tasks was not related to whether they had a pet at home or to the amount of weekly exposure to cats and/or dogs. However, given that the majority of infants had some familiarity with cats and dogs through direct or indirect experience (e.g., TV, books, and toys), this general exposure appears to be sufficient in allowing infants to draw upon their pre-existing representations of the animal–sound pairing.

Our findings extend the results of recent research demonstrating that infants’ prior experience with a set of stimuli impacts their processing of these stimuli in the experimental context (Bornstein and Mash, 2010; Hurley et al., 2010; Kovack-Lesh et al., 2014), with differences being noted between infants with and without pets. Our experiments differ, however, in an important way—that is, in our task, we did not examine infants’ processing of information over the course of the experiment but rather focused on the outcome of the task (i.e., on the mappings and the generalization of the sound property). Thus, lack of differences in the performance of infants with and without pets could reflect differences in the variables of interest. Consistent with this interpretation, there is evidence to suggest that, although prior experience influences infants’ online behavior, it does not necessarily impact infants’ categorical decisions (e.g., Bornstein and Mash, 2010). Future research could conduct a more fine-grained analysis of infants’ online behavior during property generalization tasks (e.g., by using eye-tracking methodologies) to determine whether it impacts infants’ performance.

There are a number of limitations to these studies to be noted. One pertains to the cat and dog stimuli used. That is, we opted to use cats and dogs in an attempt to assess the potential role of prior exposure to cats and dogs on infants’ property extensions. Although we collected information on whether infants had a pet and the amount of weekly exposure to cats and dogs, these variables were not related to infants’ performance in the experimental tasks. Thus, we proposed that the observed facilitative effect in the context of naturally occurring, basic-level categories stems from general exposure to these animals acquired throughout a variety of means (e.g., direct experience with a pet, exposure through books, television, and toys). Yet, the precise mechanism by which this occurs remains unclear. Future research can more directly manipulate familiarity or experience with a set of stimuli (e.g., as in Bornstein and Mash, 2010) by familiarizing one group of infants with object–property pairings and comparing their property generalizations to a group of infants without prior experience with the associations that need to be learned.

Another potential limitation of the present studies comes from the sole focus on infants’ overall looking times to determine whether infants made property extensions. Although overall looking times are widely used in infancy research, there is evidence to suggest that obtaining a more fine-grained analysis can provide new insights into infants’ performance (e.g., Kovack-Lesh et al., 2008, 2014; Hurley et al., 2010). As such, using methodologies such as eye tracking can help to provide further insight into the process of categorization and subsequent property generalizations. That is, by doing a more fine-grained analysis of infants’ looking, it is possible to assess infants’ *processing* of the stimuli during the task and to determine whether and/or how the features that infants’ attend to during learning impact their ability to make property generalization.

In closing, our results suggest that the ability to generalize properties within naturally occurring, basic-level categories is present by 11 months of age, providing a critical step in characterizing the developmental emergence of inductive reasoning. Of course, there are several questions that remain to be addressed by future research. First, it will be critical to examine how category type (i.e., animates vs. inanimates) and property type (i.e., sound properties vs. other types of properties) may influence infants’ abilities to generalize properties during the first year of life. Second, further research is needed to delineate the mechanisms by which infants make property extensions (i.e., to differentiate between reasoning-based, similarity-based, and attentional accounts). Furthermore, examining similarities and differences in processes across different types of inductive problems in infancy (i.e., word learning, probabilistic reasoning, category-based inductive reasoning) will significantly advance our understanding of key mechanisms involved.

## Data Availability Statement

The raw data supporting the conclusions of this article will be made available by the authors, without undue reservation.

## Ethics Statement

The studies involving human participants were reviewed and approved by the Conjoint Faculties Research Board at the University of Calgary (Project title: Reasoning about object categories and properties during early childhood REB16-0423). Written informed consent to participate in this study was provided by the participants’ legal guardian/next of kin.

## Author Contributions

EV conducted this research in partial fulfillment on the requirements for the Ph.D. degree, under the supervision of SG. Some of the data from this experiment were included in EV’s Ph.D. thesis, submitted to the University of Calgary. MZ and NA assisted with data collection. All authors contributed to the conceptualization, design, and analysis of the data and to the preparation of the manuscript.

## Conflict of Interest

The authors declare that the research was conducted in the absence of any commercial or financial relationships that could be construed as a potential conflict of interest.

## Footnotes

1 https://medstats.github.io/bayesfactor.html

## Supplementary Material

Supplementary Data Sheet 1 Appendix A: Pilot study.
