# Supplementary material for: Eleven-Month-Olds Link Sound Properties With Animal Categories
Source: Front Psychol. 2020 Oct 19;11:559390. doi: 10.3389/fpsyg.2020.559390 (PMC7604356; doi:10.3389/fpsyg.2020.559390)
Supplement: Supplementary Data Sheet 1 — Appendix A: Pilot study. [file Data_Sheet_1.pdf]

Appendix A: Pilot Study

To lay the foundation for our subsequent studies, we first examined whether 11-month-olds would correctly match a characteristic sound with the appropriate animal in the absence of exposure to the animal-sound pairing in the experimental context. If infants spontaneously link the characteristic sounds that cats and dogs make to their depicted referents, this would suggest that they have already formed a robust category-sound link at this age.

Eleven-month-olds were exposed to static pictures of a cat and a dog with no accompanying sound. We then tested infants in a preferential looking paradigm. Videos of a cat and a dog were presented side-by-side, while one sound played (either meowing or barking). If infants brought previous knowledge about the sounds emitted by cats and dogs to the experiment, we expected that they would look toward the target animal (i.e., the animal that matched the sound) at rates significantly greater than chance. In other words, consistent with previous work, we expected infants to spend a greater proportion of time looking to the congruent match (e.g., (e.g., Bahrick et al., 2005; Flom, Whipple, & Hyde, 2009).

Method

Participants

The final sample consisted of 32 11-month-olds. One additional infant was tested but was excluded due to experimenter error. Information on mean age, gender, parent education, and whether infants had a pet at home is included in Table 1. Infants came from homes in which English was the predominant language spoken and, although not formally assessed, infants were primarily of European descent.

Stimuli

The visual stimuli presented during the exposure phase consisted of static pictures of cats and dogs. Two exemplars from each category that differed in color were used (see Figure 7). At test, the visual stimuli consisted of animated videos of cats and dogs opening and closing their mouths in synchrony. At test, infants heard one of two real animal sounds: *meowing* (76.24 decibels (dB) and 576.46 hertz (Hz)) and *barking* (75.96 dB and 600.02 Hz).

Apparatus

Infants were tested in a soundproof, dimly lit room, and sat on their parent's lap or on a high chair. The visual stimuli were presented on a 122 cm x 91.5 cm monitor, and the auditory stimuli were played from a speaker placed directly above the monitor. Parents listened to music through headphones and were asked to have minimal interaction with their infants. The Habit X 1.0 program was used to run the experiment (Cohen, Atkinson, & Chaput, 2004). All testing sessions were recorded for later coding of infants' looking time on a frame-by-frame basis.

Procedure

Prior to the experimental task, parents were asked about infants' exposure to cats and dogs as described in the main experiments. Figure 7 provides an overview of the experimental design. Infants were exposed to static pictures of a cat and a dog presented sequentially (e.g., Orange Cat and Black Dog) for a total of 4 trials. Each trial lasted 10 seconds, and each animal was presented twice over the course of the brief exposure phase. No sound information was presented during the exposure phase. Next, infants were tested using a preferential looking paradigm. Videos of a cat and a dog were presented side-by-side (53 cm apart), while infants

heard one sound (*barking* or *meowing*). The animals faced the infants, and opened and closed their mouths in synchrony for a total of 20 seconds, alternating between periods of sound (0.5 seconds) and silence (1 second). Infants' tendency to match the sound property to its respective animal was tested twice: with the same cat and dog presented during the exposure phase (e.g., Orange Cat – Black Dog – *Bark*) and with a new category member (e.g., Gray Cat – Brown Dog – *Bark*). Thus, there were two trials assessing matching of the cat sound and two trials assessing matching of the dog sound. Infants were tested in 1 of 24 counterbalanced orders (i.e., across participants, we counterbalanced the specific cat and dog picture presented during exposure, the order in which the cat and dog pictures appeared during exposure, the side that each animal appeared on during test, and the specific order of the sound being tested). Given that our final analysis included 32 infants, eight of the orders were repeated.

Looking times for all trials were coded on a frame-by-frame basis from video to obtain a more accurate measure of infants' looking times as compared to online coding. Centre fixations were coded for the exposure trials, and left and right looks were coded for the test trials. Coders were unaware of the study purpose and hypotheses and were unable to identify the target animal during test trials (as coding was conducted with the sound turned off). Interrater reliability for 15% of the data (n = 5) was high (ICC = .98, p < .001).

Results and Discussion

We first calculated a proportion of looking score for the trials that assessed knowledge of the cat and dog sounds by dividing looking time to the target animal by the total looking time during a given trial. Next, to determine whether 11-month-olds' performance differed as a function of trial type or order, we conducted a 2 (trial type – *cat* and *dog* trials) x 2 (order – *first* vs. *second animal set*) within-subjects ANOVA. All effects were non-significant (ps > .760).

Our analysis of interest focused on infants' ability to match each sound to its respective animal. To address this question, we examined whether infants' proportion of looking to the target animal was significantly greater than chance (50%), using one-sample t-tests. First, we averaged across trials to obtain a proportion of looking score by animal type (given that the effect of order was non-significant). Two-tailed one sample t-tests indicated that 11-month-olds did not look to the target animal at rates significantly above chance for the cat (M = .52; SD = .10) or for the dog (M = .51; SD = .07) trials, t (31) = 1.27, p = .211, and t (30) = 0.83, p = .411, respectively (one infant was excluded from the analyses as they were a statistical outlier (z-score greater than 3 SD).

The above analyses indicate that 11-month-olds did not match the target sound with the target animal. These frequentist analyses, however, do not allow us to conclude that 11-month-olds do not spontaneously link the characteristic sounds that cats and dogs make to their depicted referents —only that we failed to find support for the hypothesis that they do have this knowledge (Dienes, 2014; Dienes & Mclatchie, 2018). A Bayes factor analysis was used to assess the relative strength of the evidence for null hypothesis versus the alternative hypothesis (i.e., looking to the matching animals at above chance levels; [59]), using the same criteria describe in the previous experiments. For the dog, the resulting Bayes Factor, $B_{\mathrm{H}\,(0,\,0.09)}$ = 0.33, indicates evidence for the null hypothesis. For the cat, the Bayes Factor, $B_{\mathrm{H}\,(0,\,0.09)}$ = 0.77, falls towards the support of the null hypothesis but is indeterminate.

Next, we examined whether infants' performance on trials assessing their knowledge of cat and dog sounds was related to whether or not they had a pet at home or to their average

amount of weekly exposure to a pet. We did not find differences in infants' performance on test trials as a result of pet type (e.g., infants who had pet dogs at home did not perform differently from infants without dogs at home, $p = .238$); thus, our correlations focused only on whether infants had a pet at home rather than the specific type of pet. Infants' matching of sounds on cat trials and dog trials was not correlated with whether infants had a pet at home, r (30) = -.01, $p = .951$, and, r (30) = -.33, $p = .065$, respectively. Similarly, infants' overall weekly exposure ($M = 27.38$, $SD = 29.70$; 0 – 84 hours per week) to a pet did not correlate with their performance on trials linking meowing to the cat, r (30) = .15, $p = .422$, or barking to the dog, r (30) = -.23, $p = .204$.

Eleven-month-olds did not show evidence of spontaneously matching the respective sounds to cats and dogs under the specific learning conditions of our experimental task (although we note that the Bayes Factor results for the cat trials was indeterminate). Further, infants' performance on the task was not related to whether they had a pet at home or to the amount of weekly exposure to a pet.

Appendix B

Across our studies, infants' performance on the experimental task was not related to whether they had a pet at home or to the amount of weekly exposure to cats and dogs. Here, we report the specific correlations for Experiments 1 to 3. We note that due to the small sample sizes, these analyses are considered exploratory and should be interpreted with caution.

Experiment 1

We examined whether infants' performance was related to whether they had a pet at home. Our correlations focused on whether infants had a pet at home rather than the specific type of pet, consistent with prior research suggesting that experience with a pet in general, rather than a specific type of pet, can impact infants' performance (e.g., Kovack-Lesh, Horst, & Oakes, 2008). We first calculated an overall performance score, collapsing across condition and trial type (as performance did not differ as a function of condition or trial type) and correlated that score with whether infants had a pet at home or not (yes/no). Eleven-month-olds' performance on our task was not related to whether they had a pet a home, r (53) = -.148, p = .291, or to the amount of weekly exposure to a pet, r (53) = -.246, p = .076.

Experiment 2

When presented with incongruent animal-sound pairs, 11-month-olds' proportion of looking did not differ significantly from chance for both *same* and *extension* trials. Thus, we collapsed across trial type to calculate an average score that captured infants' overall performance. Infants' performance in this group did not correlate with whether they had a pet at home, r (30) = .027, p = .889, or to the amount of weekly exposure to a pet, r (30) = .109, p = .567.

Experiment 3

When presented with novel sound-animal, 11-month-olds' proportion of looking differed significantly from chance for *same* trials *but not extension* trials. Thus, we conducted separate correlations for each trial type. Infants' performance on the Same trials did not correlate with whether they had a pet at home, r (30) = -.030, p = .877, or to the amount of weekly exposure to a pet, r (30) = -.029, p = .879. Similarly, infants' performance on the extension trials did not correlate with whether they had a pet at home, r (30) = .310, p = .095, or to the amount of weekly exposure to a pet, r (30) = -.192, p = .309.
